# Supplementary material for: The Virome of Bats Inhabiting Brazilian Biomes: Knowledge Gaps and Biases towards Zoonotic Viruses
Source: Microbiol Spectr. 2023 Jan 10;11(1):e04077-22. doi: 10.1128/spectrum.04077-22 (PMC9927472; doi:10.1128/spectrum.04077-22)
Supplement: Supplemental file 5 — Supplemental material. Download spectrum.04077-22-s0005.pdf, PDF file, 0.2 MB [file spectrum.04077-22-s0005.pdf]

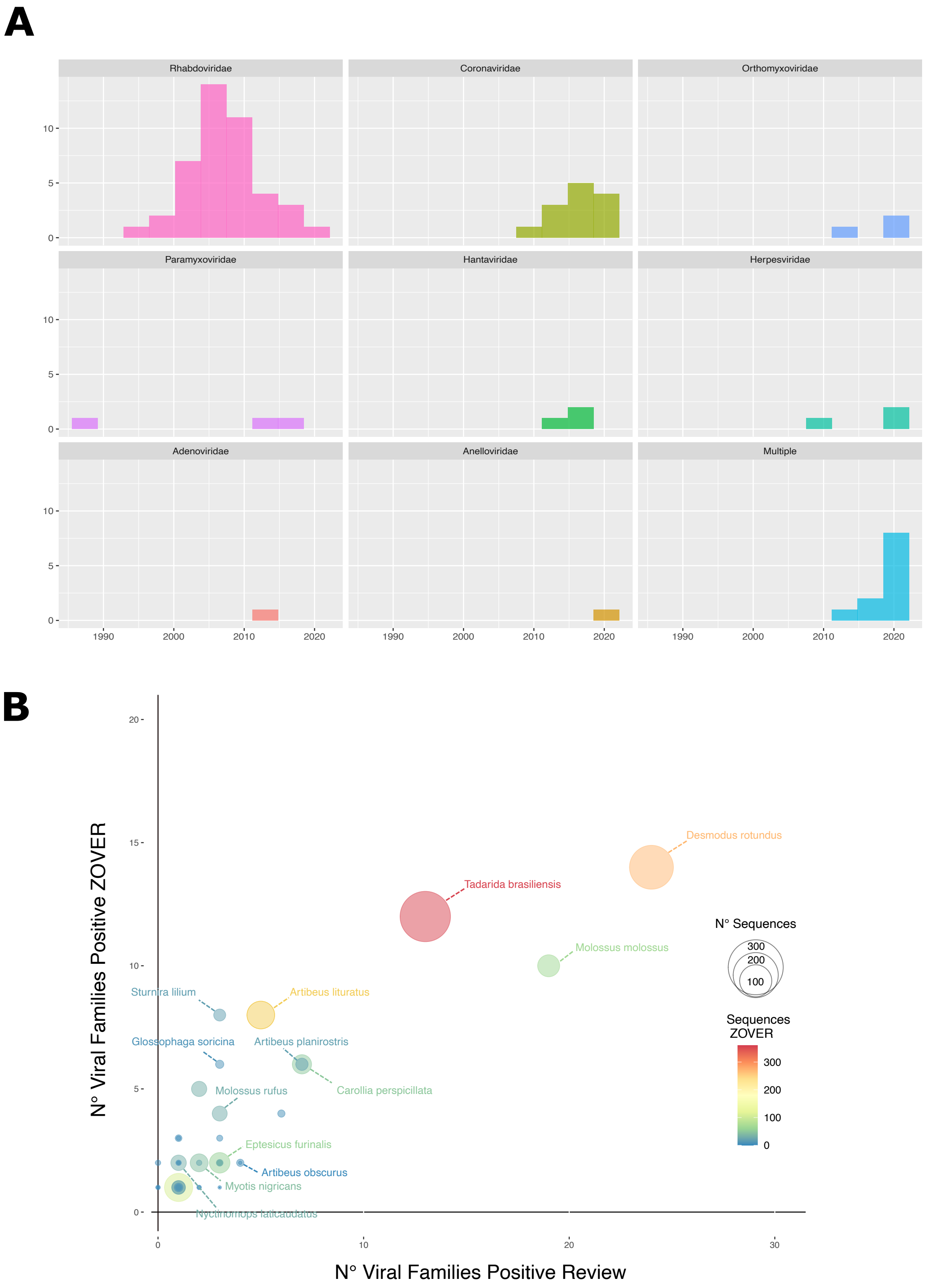

Figure S1- Distribution of studies per viral family per year (A). Correlation between the number of viral families positive in the review and ZOVER databases (B).

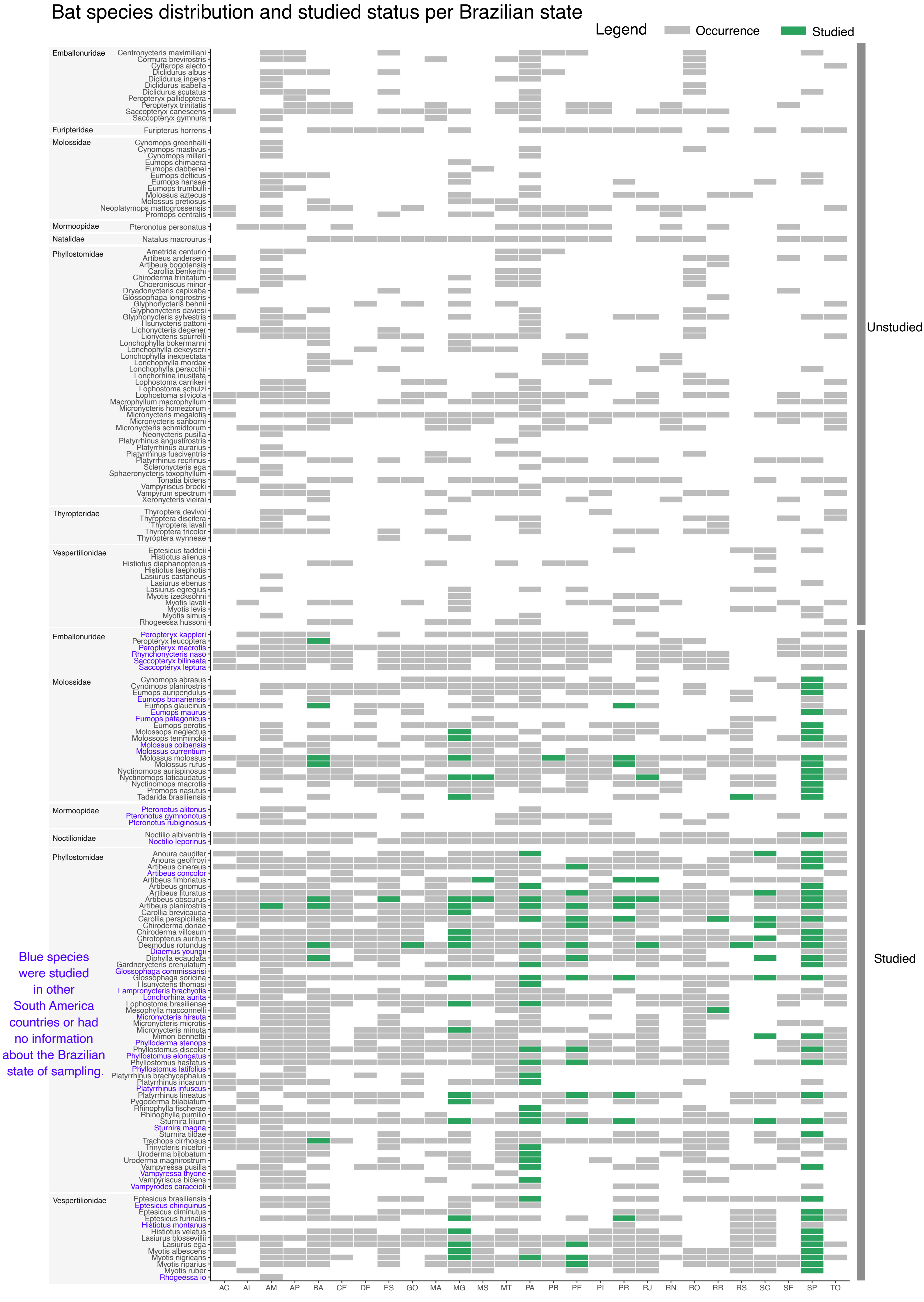

Figure S2- Bat species distribution and studied status per Brazilian state.

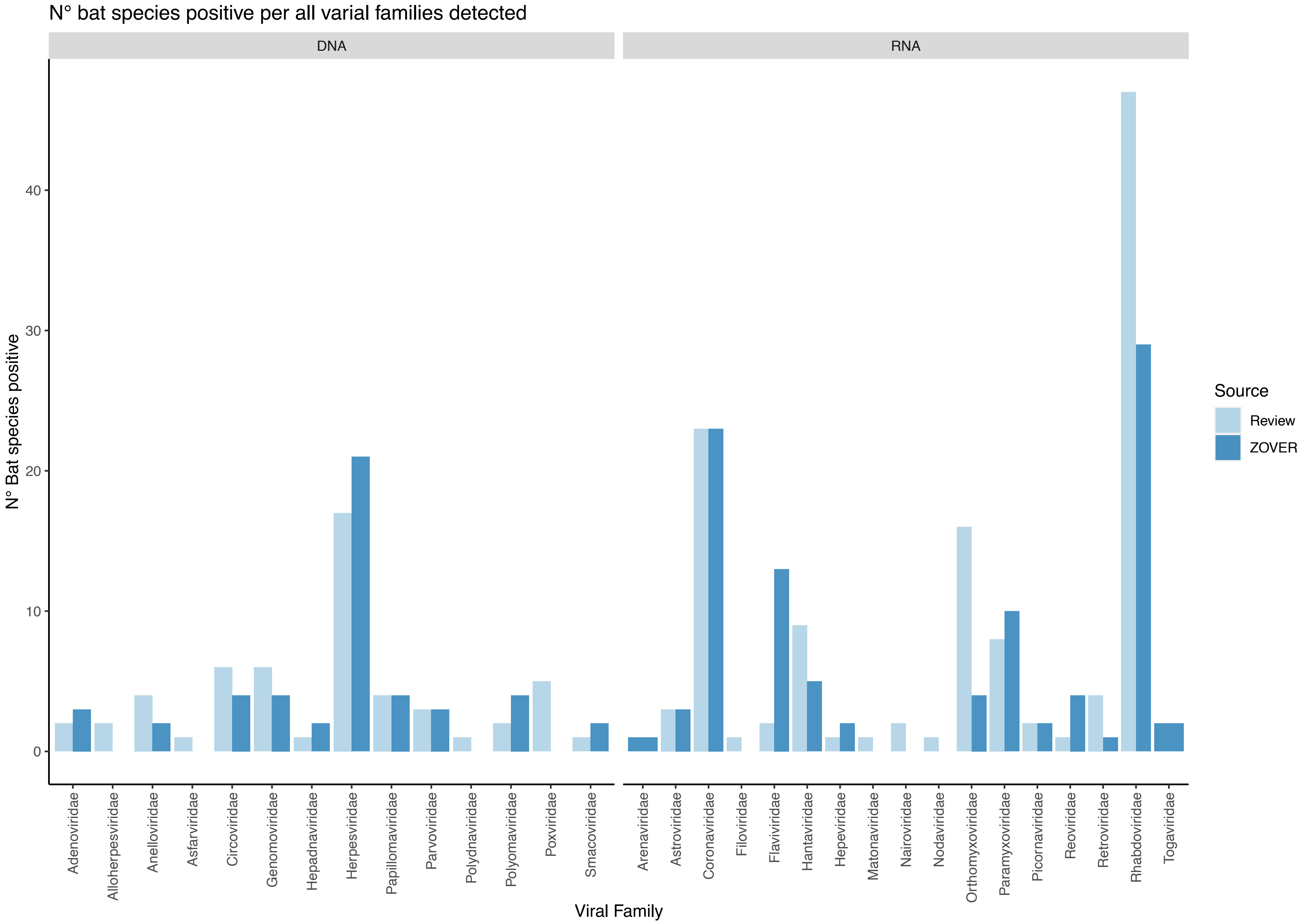

Figure S3 - Number of bat species positive per viral family in ZOVER and the Review database.

Figure S1 - Distribution of studies per viral family per year (**A**). Correlation between the number of viral families positive in the review and ZOVER databases (**B**).

Figure S2 - Bat species distribution and studied status per Brazilian state.

Figure S3 - Number of bat species positive per viral family in ZOVER and the Review database.

Supplementary File 1 - List of all manuscripts reviewed in this study including the methodologies employed for viral detection.

Supplementary File 2 - Detailed information of each manuscript data extracted.

Supplementary File 3 - Number of studies and number sequences available in ZOVER for each bat species.

Supplementary File 4 - Additional stratification of manuscrits based on number of methodologies for viral detection employed, methodology throughput, longitudinal sampling and sample type investigated.
